# Mean pulmonary arterial pressure as a prognostic indicator in connective tissue disease associated with interstitial lung disease: a retrospective cohort study

**DOI:** 10.1186/s12890-016-0207-3

**Published:** 2016-04-19

**Authors:** Kota Takahashi, Hiroyuki Taniguchi, Masahiko Ando, Koji Sakamoto, Yasuhiro Kondoh, Naohiro Watanabe, Tomoki Kimura, Kensuke Kataoka, Atsushi Suzuki, Satoru Ito, Yoshinori Hasegawa

**Affiliations:** Department of Respiratory Medicine, Nagoya University Graduate School of Medicine, Nagoya, Aichi Japan; Department of Respiratory Medicine and Allergy, Tosei General Hospital, 160 Nishioiwake-cho, Seto, Aichi 489-8642 Japan; Center for Advanced Medicine and Clinical Research, Nagoya University Hospital, Nagoya, Aichi Japan

**Keywords:** Interstitial lung diseases, Pulmonary hypertension, Connective tissue disease, Mean pulmonary arterial pressure, Interstitial pneumonia, Prognosis

## Abstract

**Background:**

Pulmonary hypertension (PH) can develop in connective tissue disease associated interstitial lung disease (CTD-ILD), and contributes to increased morbidity and mortality. However, except for systemic sclerosis and mixed connective tissue disease, the impact of mean pulmonary arterial pressure (MPAP) on survival in CTD-ILD has not been sufficiently elucidated. We hypothesized that pulmonary arterial pressure may be a prognostic factor in CTD-ILDs regardless of the kind of CTD.

**Methods:**

We evaluated the survival impact of MPAP, which is measured using right heart catheterization, on survival of patients with CTD-ILD with various CTD backgrounds. We retrospectively analyzed data of consecutive CTD-ILD patients undergoing a pulmonary function test and right-heart-catheterization at the initial evaluation.

**Results:**

We studied 74 patients (33 men and 41 women, mean age 62.8 ± 9.6, 24 with rheumatoid arthritis, 14 with systemic sclerosis, 14 with polymyositis/dermatomyositis, 11 with primary Sjögren’s syndrome, and 11 with other diagnoses). Six patients exhibited pulmonary hypertension (MPAP ≥ 25 mmHg), and 16 (21.6 %) had mild elevation of MPAP (≥20 mmHg). The mean MPAP was 17.2 ± 5.5 mmHg. We did not observe a significant difference in MPAP among various CTDs. A univariate Cox proportional hazard model showed that MPAP has a significant impact on survival, while the type of CTD did not contribute to survival in our cohort. A multivariate Cox proportional hazard model showed MPAP (HR = 1.087; 95 % CI 1.008–1.172; *p* = 0.030) to be the sole independent determinant of survival.

**Conclusions:**

Mild elevation of MPAP is relatively common in CTD-ILD patients with various CTD backgrounds. A higher MPAP at the initial evaluation was a significant independent predictor of survival in CTD-ILD. MPAP evaluation provides additional information on disease status and will help physicians predict mortality in CTD-ILD.

## Background

Patients with pulmonary hypertension (PH) in association with underlying interstitial lung disease (ILD) have worse survival rates than those with ILD alone [1–3]. PH in patients with systemic sclerosis (SSc) and ILD (SSc-ILD) further worsens survival, but there are few data regarding outcomes in SSc-ILD patients with PH compared with those with the other connective tissue disease (CTD) associated ILD [4, 5].

Recently, the importance of mild elevation in pulmonary arterial pressure has been recognized in patients with several ILDs. It has been demonstrated that a mean pulmonary arterial pressure (MPAP) between 21 and 24 mmHg is clinically relevant and affects patient outcome in idiopathic pulmonary fibrosis (IPF) [3, 6]. Furthermore, mild elevation of MPAP is also implicated in SSc patients [7]. Fischer et al. proposed “lung-dominant CTD” (LD-CTD) as a concept for classifying the subset of patients with ILD who have clinical features suggesting an associated CTD, but whose features fall short of a clear diagnosis of CTD under the current rheumatologic classification systems [8]. More recently, we elucidated that MPAP ≥ 20 mmHg affects patient outcome in LD-CTD [9].

To date, little information is available regarding prognostic factors in ILDs with various CTD backgrounds. We hypothesized that pulmonary arterial pressure may be a prognostic factor in CTD-ILDs in spite of the kind of CTD. The aim of this study was to determine whether MPAP predicts survival in CTD-ILD patients whose disease background, pulmonary function test, and right heart catheterization (RHC) data are available. The significance of mild MPAP elevation (≥20 mmHg) was also explored [9].

## Methods

### Subjects

**Fig. 1:**
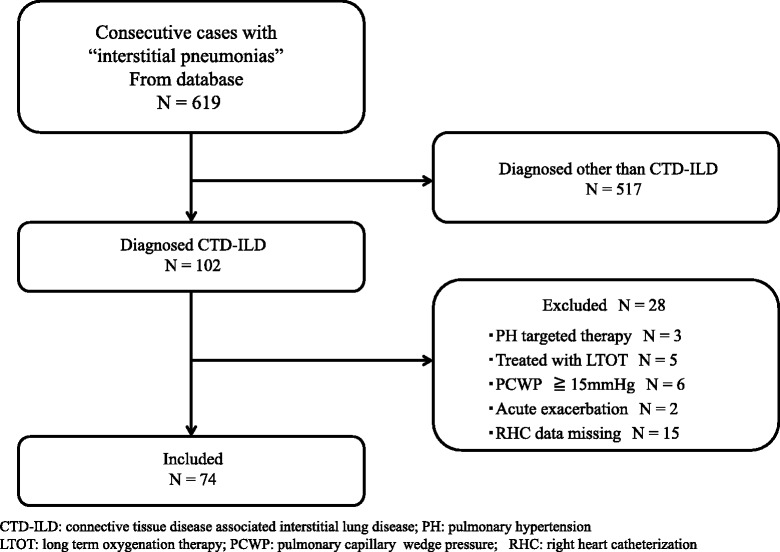
Criteria for screening and inclusion of patients in the study. CTD-ILD: connective tissue disease associated interstitial lung disease; PH: pulmonary hypertension; LTOT: long term oxygenation therapy; PCWP: pulmonary capillary wedge pressure; RHC: right heart catheterization

In this study, we retrospectively analyzed our ILD patient database, which registers the results of systematic evaluations undergone at the time of their initial consultation with our department at Tosei General Hospital (Aichi, Japan). Of 619 records of patients with ILD examined between July 2007 and June 2012, 102 were diagnosed with CTD-ILD. Twenty-eight patients were excluded from the study for the following reasons: (1) they had been treated with PH targeted therapy, (2) evaluation was done using supplemental oxygen, (3) they were suffering from an unstable disease, such as acute exacerbation, infection, or heart failure, or (4) RHC was not performed within 3 months of initial evaluation or RHC data was missing. Finally, we reviewed the charts of 74 stable CTD-ILD patients who underwent RHC for evaluation during this period (Fig. 1).

PH was assessed by RHC and defined as a pulmonary arterial pressure ≥ 25 mmHg. In this study, we also adopted MPAP ≥ 20 mmHg as a cut-off for predicting the prognosis based on our previous study of lung-dominant CTD patients [9].

This study was approved by the Tosei General Hospital Institutional Review Board (IRB No. 483). Patient records and information were anonymized and de-identified prior to analysis. The diagnosis of ILD was made clinically by high-resolution CT (HRCT) [10]. Pathological diagnoses of 33 patients were confirmed by surgical lung biopsy but did not meet the criteria for the clinical diagnosis of IPF [11] or other definite ILDs. The clinical diagnosis of CTD for each subject, which was made according to established criteria, was also classified and recorded as : SSc, rheumatoid arthritis (RA), systemic lupus erythematosus (SLE), polymyositis/dermatomyositis (PM/DM), mixed connective tissue disease (MCTD), or Sjögren’s syndrome (SjS) (all according to established criteria) [12–18].

### Measurements

We recorded patients’ demographic characteristics, results of pulmonary function tests (PFTs), PaO_2_ assessed by arterial blood gas analysis, diagnosis of CTD, medication record, and hemodynamics measured by RHC, retrospectively. All patients underwent spirometry (CHESTAC-55 V; Chest, Tokyo, Japan), according to the method described in the ATS 1994 update [19]. Single-breath diffusing lung capacity of carbon monoxide (DL_CO_) was also measured (CHESTAC-55 V). The values for forced vital capacity (FVC) and DL_CO_ were related to % predicted values [20]. RHC was performed using a Swan-Ganz catheter percutaneously inserted into either a cubital vein or femoral vein under normal conditions as the patient breathed room air. We did not perform a vasoreactivity test. MPAP was measured with the catheter tip placed within the right main trunk of the pulmonary artery. Cardiac output was measured by a thermodilution method.

### Statistical analysis

Survival status was ascertained in September 2014. Among 74 eligible patients, 15 deaths were observed. This study had at least 70 % statistical power to detect a difference of 40 % in 5-year survival rates (80 % vs 40 %) between patients with MPAP < 20 mmHg and those with MPAP ≥ 20 mmHg at the 5 % significance level. Continuous variables were summarized as the mean ± SD or the median and range and compared using Student’s *t*-test or Wilcoxon’s rank sum test where appropriate. Categorical variables were compared using the chi-square statistic. Pearson’s correlation coefficient was used to evaluate the correlations between MPAP and clinical values. If either variable had a non-normal distribution, correlations were calculated using Spearman’s correlation test. Univariate Cox’s proportional hazard models were used to examine the association of selected variables with survival. A multivariate analysis was used to elucidate independent prognostic factors. Variables with p < 0.15 were used for entry into the model. To avoid multicolinearity, only one of the highly correlated variables (coefficient of correlation ≥ 0.9) was used in the multivariate model, if more than one was present. Using the methods of Kaplan-Meier and the log-rank test, we studied the impact of variables on survival. *P* values less than 0.05 were considered significant. All analyses were performed using a statistical software package (SPSS, version 19.0; SPSS, Inc., Chicago, IL).

## Results

The baseline demographic and clinical characteristics of the 74 patients are summarized in Table 1. The numbers of patients with RA, SSc, PM/DM, SjS, MCTD, SLE, and overlap syndrome were 24 (32.4 %), 14 (18.9 %), 14 (18.9 %), 11 (14.9 %), 5 (6.8 %), 1 (1.4 %), and 5 (6.8 %), respectively. Thirty three patients (44.6 %) underwent surgical lung biopsy. Forty-seven patients (65.2 %) had decreases in diffusing capacity. Six patients (8.1 %) had PH (MPAP ≥ 25 mmHg): three with PM/DM and one each with RA and MCTD, respectively. Sixteen patients (21.6 %) had MPAP ≥ 20 mmHg: 7 with RA, 4 with PM/DM, two each with SSc and MCTD, and one with overlap syndrome, respectively. The patients with RA, SSc, PM/DM, SjS, MCTD, SLE, and overlap syndrome had a mean ± SD MPAP of 17.0 ± 5.0 mmHg, 16.3 ± 4.1 mmHg, 17.6 ± 6.0 mmHg, 16.1 ± 5.7 mmHg, and 23.5 ± 8.7 mmHg, 19.0 mmHg, and 16.3 ± 3.7 mmHg, respectively. Twenty seven patients (36.5 %) received pharmacological treatment for CTD or ILD at the RHC evaluation. Twenty three patients (31.1 %) were treated with oral corticosteroids. Ten patients (13.5 %) were treated with an immunosuppressive agent (cyclosporin:tacrolimus:cyclophosphamide:methotrexate 6:2:1:1). Four patients (5.4 %) were treated with beraprost sodium orally for Raynaud’s phenomenon. No patient was treated with PH targeted therapy and antithrombotic agents for PH. No patient had received long-term oxygen therapy (LTOT) at the time of RHC evaluation.

**Table 1:** Baseline characteristics of all patients with CTD-ILD

Variable		Range
Sex, M/F	33/41	
Age, years	63.5 ± 1.1	38–78
BMI, kg/m^2^	23.0 ± 4.0	13.7–35.3
Smoking status, ever/never	31/43	
CTD		
RA/SSc/PMDM/SjS/MCTD/	24/14/14/11/5	
SLE/overlap syndrome	1/5	
Surgical lung biopsy, yes/no	33/41	
Corticosteroids/immunosuppressant	23/10	
FVC, % predicted	81.0 ± 19.1	38.3–127.0
DLco, % predicted	57.1 ± 19.2	22.6–113.6
RV, % predicted	81.2 ± 31.5	17.9–181.9
TLC, % predicted	80.3 ± 18.3	47.9–138.6
PaO_2,_ mmHg	80.8 ± 9.9	60.2–103.2
A-a gradient, mmHg	20.2 ± 11.5	−3.8–38.1
MPAP, mmHg	17.2 ± 5.5	8–36
CI, l/min/m^2^	3.59 ± 0.77	1.89–5.18
PVR, Wood units	127.3 ± 59.9	34.0–406.0
PVRI, Wood units/m^2^	223.5 ± 102.1	52.0–606.0

Age is presented as median ± SE. Data are presented as mean ± SD unless otherwise noted. BMI: body mass index; RA: rheumatoid arthritis; SSc: systemic sclerosis; PM/DM: polymyositis/dermatomyositis; SjS: Sjögren’s syndrome; MCTD: mixed connective tissue disease; SLE: systemic lupus erythematosus; FVC: forced vital capacity; DLco: diffusing capacity of the lung for carbon monoxide; RV: residual volume, TLC: total lung capacity, PaO_2_: arterial oxygen tension; A-a gradient: alveolar-arterial O_2_ tension gradient, MPAP: mean pulmonary arterial pressure; CI: cardiac index; PVR: pulmonary vascular resistance; PVRI: pulmonary vascular resistance index. *N* = 74 except for DLco (*n* = 72)

In terms of survival analysis, fifteen patients (20.3 %) died during the mean observation period of 44.4 ± 2.2 months: six deaths were due to advanced respiratory failure, four due to acute exacerbation, three due to malignant disease, one due to multiple organ failure, and one due to neuromuscular disease. The univariate Cox regression model (Table 2) demonstrated that MPAP (*p* = 0.014) had a statistically significant impact on survival. Sex (*p* = 0.106), smoking status (*p* = 0.102), and RV, % predicted (*p* = 0.102) did not reach statistical significance. The multivariate analysis including all relevant variables with *p* values < 0.15 in univariate analysis showed that only MPAP (HR = 1.087; 95 % CI1.008–1.172, *p* = 0.030) was significantly correlated with survival (Table 3). Patients with PH (MPAP ≥ 25 mmHg) had a significantly worse prognosis than those without PH (HR = 3.995; 95 % CI 1.267–12.60, *p* = 0.018). When we conducted further analysis of survival time to respiratory specific death (including only deaths due to respiratory failure), MPAP still significantly correlated with survival (HR = 1.154; 95 % CI1.033–1.289, *p* = 0.011). In addition, there was no association between any CTD background and survival (SSc and MCTD vs. the others: HR = 1.186; 95 % CI0.333–4.223, *p* = 0.792, RA vs. the others: HR = 0.530; 95 % CI0.192–1.465, *p* = 0.221, and PM/DM vs. the others: HR = 1.739; 95 % CI0.392–7.714, *p* = 0.467).

**Table 2:** Results of univariate Cox proportional hazard model for mortality

Variable	HR	95 % CI	*P* value
Age, years	1.018	0.958–1.082	0.570
Sex, M/F	2.366	0.833–6.721	0.106
BMI, kg/m^2^	0.956	0.841–1.087	0.493
Smoking status, ever/never	2.377	0.842–6.706	0.102
SSc + MCTD diagnosis vs. others	1.186	0.333–4.223	0.792
RA diagnosis vs. others	0.530	0.192–1.465	0.221
PM/DM diagnosis vs. others	1.739	0.392–7.714	0.467
Corticosteroids and/or immunosuppressant use, yes/no	0.666	0.236–1.885	0.444
FVC, % predicted	0.998	0.972–1.025	0.889
DL_CO_, % predicted	0.982	0.951–1.013	0.252
RV, % predicted	1.010	0.998–1.023	0.102
TLC, % predicted	1.005	0.979–1.033	0.699
PaO_2_, mmHg	0.978	0.928–1.032	0.420
A-a gradient, mmHg	1.025	0.982–1.071	0.259
MPAP, mmHg	1.093	1.018–1.175	0.014
CI, l/min/m^2^	0.651	0.304–1.392	0.268
PVR, Wood units	1.005	0.998–1.011	0.189
PARI, Wood units/m^2^	1.003	0.999–1.008	0.156

HR: hazard ratio; CI: confidence interval (see Table 1 for other definitions)

**Table 3:** Results of multivariate Cox proportional hazard model for mortality

Variable	HR	95 % CI	*P* value
Sex, M/F	1.747	0.465–6.563	0.409
Smoking, ever/never	1.740	0.467–6.482	0.409
RV, % predicted	1.007	0.993–1.021	0.322
MPAP, mmHg	1.087	1.008–1.172	0.030

Variables used were *P* value < 0.15 in univariate analysis (Table 2)

**Fig. 2:**
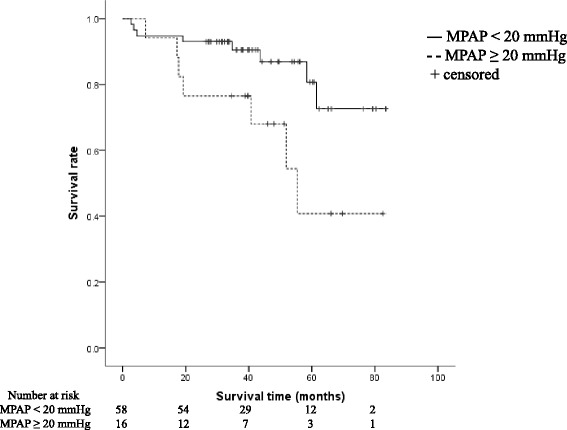
Kaplan-Meier curves for survival according to MPAP. Survival curves of patients with MPAP ≥ 20 mmHg and MPAP < 20 mmHg were compared and tested with log-rank statistics (*p* = 0.023)

To evaluate the significance of mild elevation of MPAP with a cutoff point of 20 mmHg for survival, the following analysis was performed. As shown in Table 4, patients were stratified according to MPAP with the cutoff point of 20 mmHg, and their baseline characteristics and physiology were compared. Male sex and a history of smoking were more frequent in patients with MPAP ≥ 20 mmHg. Figure 2 shows a Kaplan-Meier curve that reveals significantly worse survival among patients whose MPAP was ≥ 20 mmHg than among those whose MPAP was < 20 mmHg (log-rank test *p* = 0.023).

**Table 4:** Baseline characteristics and physiology of patients with and without MPAP ≥ 20 mmHg

Variable	MPAP < 20 mmHg	MPAP ≥ 20 mmHg	*P* value
	(*N* = 58)	(*N* = 16)	
Sex, M/F	24/34	9/7	<0.001
Age, years	62.9 ± 9.0	61.9 ± 11.4	0.777
BMI	22.7 ± 3.9	23.8 ± 4.3	0.270
Smoking, ever/never	22/36	9/7	<0.001
FVC, % predicted	82.5 ± 19.3	76.0 ± 17.4	0.318
DL_CO_, % predicted	58.8 ± 17.9	51.2 ± 23.0	0.092
RV, % predicted	82.6 ± 30.9	76.3 ± 34.2	0.867
TLC, % predicted	82.6 ± 18.9	72.5 ± 13.8	0.343
PaO_2_, mmHg	81.3 ± 10.2	78.6 ± 8.9	0.627
A-a gradient, mmHg	18.7 ± 11.6	25.1 ± 10.0	0.182
MPAP, mmHg	14.9 ± 2.8	24.9 ± 4.3	<0.001
Cardiac index, l/min/m^2^	3.36 ± 0.69	3.49 ± 0.79	0.458
PVRI, Wood units/m^2^	188.6 ± 73.9	258.2 ± 115.6	<0.001

Data are presented as means ± SD or numbers

*N* = 74 except for DL_CO_ (*N* = 72)

## Discussion

This is the first study, to our knowledge, to report the impact of MPAP on survival in patients of ILD with various backgrounds of CTD. We also elucidated the frequency of mild elevation of pulmonary artery pressure in this population. This study included patients with milder pulmonary function impairment (mean FVC 81.0 %, mean DLco 57.1 %) and lower MPAP (mean MPAP 17.2 mmHg) than the subjects of previous studies of IPF and SSc [7, 8]. However, a higher MPAP was shown to be an independent prognostic predictor in this population. This result suggests the importance of evaluating MPAP at an early stage in patients with CTD-ILD, and not limiting it to patients with advanced disease. Furthermore, it was shown that CTD-ILD patients with MPAP ≥ 20 mmHg had a significantly lower survival rate than those with MPAP < 20 mmHg by a log-rank test. Suzuki et al. [9] reported similar results on survival for lung-dominant CTD using the same cutoff level. Our study also demonstrated that MPAP ≥ 20 mmHg was closely associated with prognosis in CTD-ILD, which is consistent with the result in lung-dominant -CTD.

The pathogenesis of PH is likely to be complex and driven by multiple mechanisms [21]. As shown in Table 4, patients with MPAP ≥ 20 mmHg were more frequently found to have a smoking history, although we did not find a significant relationship between smoking and survival (*p* = 0.102). Notably, PH had a higher prevalence in cases of combined pulmonary fibrosis and emphysema (CPFE). These patients had clinical fibrosis associated with emphysema and smoking status [22]. Recent work has suggested that cigarette smoking plays a role in PH through inducing oxidative stress in human and animal models [23]. In addition, we have described the relationship between smoking and mild elevation of MPAP in mild IPF [6]. Our results indicate that smoking may play a role in the development of PH in CTD-ILD. Further investigation will be required to determine whether this is the case.

SSc generally represents the main CTD associated with PH. Additionally, PH occurs in 19–53 % of patients with MCTD [24, 25]. In our study, PH and MPAP ≥ 20 mmHg were frequently observed in ILD associated other types of CTD, such as RA (7 of 24, 29.2 %) and PM/DM (4 of 14, 28.6 %) in spite of excluding patients with hypoxemia. Interestingly, the prognosis of ILD patients with SSc or MCTD is not worse than those with the other CTDs. Additionally, in the case with MPAP ≥ 25 mmHg and ≥ 20 mmHg, similar results were confirmed. Launay et al. [26] reported that PH-complicated SSc was frequently detected in patients both with and without ILD. However, little is known about the prevalence and prognosis in ILD associated PH with the other CTD backgrounds. We speculate that the prevalence of PH may have clinical importance also in other CTD-ILDs such as ILD with RA and PM/DM. Therefore, it is recommended that physicians who diagnose ILD with various CTD backgrounds be careful about the elevation of pulmonary arterial pressure at the initial evaluation.

In this study, twenty three patients (31.1 %) were treated with oral corticosteroids. Ten patients (13.5 %) were treated with immunosuppressive agents. CTD-ILD patients tend to be treated with immunosuppressive therapies, although there have been few systematic and prospective studies [27, 28]. A previous study suggested that some CTD-PH patients could also benefit from immunosuppressive therapies [29]. However, in our study the therapy was not significantly associated with survival in CTD-ILD (*p* = 0.444).

Our study has several limitations. First, it is a small retrospective review at a single center, which included only patients without severe hypoxemia. Therefore, it is difficult to generalize from the result to the composition of CTD-ILD patients with severe disease status. Further large studies are needed to confirm our results. Second, we did not evaluate two clinical variables that can be confounding factors: vasoreactivity and nocturnal hypoxemia. When several statistical variables are addressed in such a small study, one wonders whether several statistical variables are truly significant or just a result of multiple comparisons. A vasoreactivity test is recommended for PAH to confirm the efficacy of calcium channel blockers. However, it is not recommended for other PH groups [3]. Therefore, further studies are needed to evaluate the significance of the vasoreactivity test for CTD-ILD. Additionally, we did not evaluate nocturnal hypoxemia. Minai et al. reported that nocturnal hypoxia is common in PAH and a risk factor for advanced pulmonary hypertension [30]. Therefore, nocturnal hypoxemia might be a prognostic factor in CTD-ILD. Additional studies to elucidate the impact of nocturnal desaturation on CTD-ILD are needed. Third, our results may be subject to treatment bias. Four patients were treated with beraprost for Raynaud’s phenomenon (not for PH), which may affect MPAP. In addition, systemic steroids and immunosuppressants affect vascular remodeling and inflammatory/autoimmune pathogenesis. It has not been elucidated whether systemic steroids and immunosuppressants affect the prognosis and pulmonary arterial pressure of patients with CTD-ILD [5]. Because additional analysis eliminating these cases did not alter any conclusion of the current study, we believe this bias did not skew our results seriously. Admittedly, further study is needed to identify whether the therapy affects MPAP and survival. Third, in some cases, the causes of death were not directly related to the patients’ CTD or ILD. However, a similar result was observed in the analysis excluding the cases that were related to non-pulmonary death. To recognize the effect of mild MPAP elevation on survival rates, further clinical studies need to be conducted with a larger population and over a longer observation period.

## Conclusions

We demonstrated that MPAP has a statistically significant impact on survival and that mild elevation of MPAP is relatively common in CTD-ILD patients with various CTD backgrounds. MPAP evaluation provides additional information on disease status and will help physicians to predict mortality in CTD-ILD.

## Abbreviations

A-a gradient: alveolar-arterial O_2_ tension gradient
CTD: connective tissue disease
CTD-ILD: connective tissue disease associated interstitial lung disease
DLco: diffusing lung capacity of carbon monoxide
FVC: forced vital capacity
ILD: interstitial lung disease
IPF: idiopathic pulmonary fibrosis
LTOT: long-term oxygen therapy
MCTD: mixed connective tissue disease
MPAP: mean pulmonary arterial pressure
PAH: pulmonary arterial hypertension
PaO_2_: arterial oxygen tension
PCWP: pulmonary capillary wedge pressure
PH: Pulmonary hypertension
PM/DM: polymyositis/dermatomyositis
PVR: pulmonary vascular resistance
PVRI: pulmonary vascular resistance index
RA: rheumatoid arthritis
RHC: right heart catheterization
RV: residual volume
SjS: Sjögren’s syndrome
SLE: systemic lupus erythematosus
SSc: systemic sclerosis
TLC: total lung capacity

## References

[1] Mej’ıa M, Carrillo G, Rojas-Serrano J, Estrada A, Sua’rez T, Alonso D (2009). Idiopathic pulmonary fibrosis and emphysema: decreased survival associated with severe pulmonary arterial hypertension. Chest.

[2] Patel NM, Lederer DJ, Borczuk AC, Kawut SM (2007). Pulmonary hypertension in idiopathic pulmonary fibrosis. Chest.

[3] Seeger W, Adir Y, Barberà JA, Champion H, Coghlan JG, Cottin V (2013). Pulmonary hypertension in chronic lung diseases. J Am Coll Cardiol.

[4] Mathai SC, Hummers LK, Champion HC, Wigley FM, Zaiman A, Hassoun PM (2009). Survival in pulmonary hypertension associated with the scleroderma spectrum of diseases: impact of interstitial lung disease. Arthritis Rheum.

[5] Fischer A, du Bois R (2012). Interstitial lung disease in connective tissue disorders. Lancet.

[6] Kimura M, Taniguchi H, Kondoh Y, Kimura T, Kataoka K, Nishiyama O (2013). Pulmonary hypertension as a prognostic indicator at the initial evaluation in idiopathic pulmonary fibrosis. Respiration.

[7] Valerio CJ, Schreiber BE, Handler CE, Denton CP, Coghlan JG (2013). Borderline mean pulmonary artery pressure in patients with systemic sclerosis: transpulmonary gradient predicts risk of developing pulmonary hypertension. Arthritis Rheum.

[8] Fischer A, West SG, Swigris JJ, Brown KK, du Bois RM (2010). Connective tissue disease-associated interstitial lung disease: a call for clarification. Chest.

[9] Suzuki A, Taniguchi H, Watanabe N, Kondoh Y, Kimura T, Kataoka K (2014). Significance of pulmonary arterial pressure as a prognostic indicator in lung-dominant connective tissue disease. PLoS One.

[10] Latsi PI, du Bois RM, Nicholson AG, Colby TV, Bisirtzoglou D, Nikolakopoulou A (2003). Fibrotic idiopathic interstitial pneumonia: the prognostic value of longitudinal functional trends. Am J Respir Crit Care Med.

[11] Raghu G, Collard HR, Egan JJ, Martinez FJ, Behr J, Brown KK, et al. An official ATS/ERS/JRS/ALAT statement: idiopathic pulmonary fibrosis: evidence-based guidelines for diagnosis and management. Am J Respir Crit Care Med. 2011;183:788–824. 10.1164/rccm.2009-040GL PMC5450933 21471066

[12] American Rheumatism Association. Preliminary criteria, for the, classification of, systemic sclerosis. (scleroderma). Subcommittee for scleroderma criteria of the American Rheumatism Association Diagnostic and Therapeutic Criteria Committee. Arthritis Rheum. 1980;23:581–90. 10.1002/art.1780230510 7378088

[13] Arnett FC, Edworthy SM, Bloch DA, McShane DJ, Fries JF, Cooper NS (1988). The American Rheumatism Association 1987 revised criteria for the classification of rheumatoid arthritis. Arthritis Rheum.

[14] Tan EM, Cohen AS, Fries JF, Masi AT, McShane DJ, Rothfield NF (1982). The 1982 revised criteria for the classification of systemic lupus erythematosus. Arthritis Rheum.

[15] Hochberg MC (1997). Updating the American College of Rheumatology revised criteria for the classification of systemic lupus erythematosus. Arthritis Rheum.

[16] Bohan A, Peter JB, Bowman RL, Pearson CM (1977). Computer-assisted analysis of 153 patients with polymyositis and dermatomyositis. Medicine (Baltimore).

[17] Kasukawa R, Sharp GC (1987). Mixed Connective Tissue Diseases and Antinuclear Antibodies.

[18] Vitali C, Bombardieri S, Moutsopoulos HM, Balestrieri G, Bencivelli W, Bernstein RM (1993). Preliminary criteria for the classification of Sjogren’s syndrome. Results of a prospective concerted action supported by the European Community. Arthritis Rheum.

[19] American Thoracic Society. Standardization of spirometry, 1994 update: American Thoracic Society. Am J Respir Crit Care Med. 1995;152:1107–36. 10.1164/ajrccm.152.3.7663792 7663792

[20] Committee of Respiratory Physiology in Japanese Respiratory Society. Guideline of respiratory function tests-spirometry, flow-volume curve, diffusion capacity of the lung. Nihon Kokyuki Gakkai Zasshi. 2004;suppl:1–56. 15565748

[21] Mathai SC, Hassoun PM (2012). Pulmonary arterial hypertension in connective tissue diseases. Heart Fail Clin.

[22] Cottin V, Le Pavec J, Prevot G, Mal H, Humbert M, Simonneau G (2010). Pulmonary hypertension in patients with combined pulmonary fibrosis and emphysema syndrome. Eur Respir J.

[23] Wright JL, Zhou S, Churg A (2012). Pulmonary hypertension and vascular oxidative damage in cigarette smoke exposed eNOS(−/−) mice and human smokers. Inhal Toxicol.

[24] Burdt MA, Hoffman RW, Deutscher SL, Wang GS, Johnson JC, Sharp GC (1999). Long-term outcome in mixed connective tissue disease: longitudinal clinical and serologic findings. Arthritis Rheum.

[25] Sullivan WD, Hurst DJ, Harmon CE, Esther JH, Agia GA, Maltby JD (1984). A prospective evaluation emphasizing pulmonary involvement in patients with mixed connective tissue disease. Medicine (Baltimore).

[26] Launay D, Mouthon L, Hachulla E, Pagnoux C, de Groote P, Remy-Jardin M (2007). Prevalence and characteristics of moderate to severe pulmonary hypertension in systemic sclerosis with and without interstitial lung disease. J Rheumatol.

[27] Vij R, Strek ME (2013). Diagnosis and treatment of connective tissue disease associated interstitial lung disease. Chest.

[28] Watanabe N, Sakamoto K, Taniguchi H, Kondoh Y, Kimura K, Kataoka K (2014). Efficacy of combined therapy with cyclosporine and low-dose prednisolone in interstitial pneumonia associated with connective tissue disease. Respiration.

[29] Sanchez O, Sitbon O, Jais X, Simonneau G, Humbert M (2006). Immunosuppressive therapy in connective tissue diseases-associated pulmonary arterial hypertension. Chest.

[30] Minai OA, Pandya CM, Golish JA, Avecillas JF, McCarthy K, Marlow S (2007). Predictors of nocturnal oxygen desaturation in pulmonary arterial hypertension. Chest.

